# Genetic Diversity of Barley Accessions from East Asia for Greenbug Resistance

**DOI:** 10.3390/plants12223797

**Published:** 2023-11-08

**Authors:** Evgeny E. Radchenko, Renat A. Abdullaev, Daria E. Akimova, Irina N. Anisimova

**Affiliations:** N.I. Vavilov All-Russian Institute of Plant Genetic Resources, 190000 St. Petersburg, Russia; abdullaev.1988@list.ru (R.A.A.); veronikadari@yandex.ru (D.E.A.); irina_anisimova@inbox.ru (I.N.A.)

**Keywords:** barley, landraces, screening, *Schizaphis graminum*, virulence, plant resistance, genes for resistance

## Abstract

The greenbug, *Schizaphis graminum*, is a dangerous pest of barley and other grain crops in the south of Russia. An effective and environmentally friendly way to control this insect is to cultivate resistant varieties. The differential interaction between the phytophage and host plants necessitates the search for new donors of resistance. Seven hundred and seventy-eight accessions of barley from East Asian countries (313 from China, 450 from Japan, and 15 from Nepal) were evaluated for greenbug resistance. The Krasnodar population of the insect and clones isolated from it were used in the experiments. Forty heterogeneous accessions were identified, in which plants with a high level of resistance to the aphid were found. As a result of damage assessment by the 108 *S. graminum* clones of 11 lines selected from heterogeneous accessions, 52 insect virulence phenotypes were identified. Experiments with aphid test clones showed that all 11 lines possess diverse greenbug resistance alleles, which differ from the previously identified *Rsg1*, but their efficiency is low. The frequency of clones virulent to ten lines and the cultivar Post (a carrier of the *Rsg1* gene) varies from 60.4% to 98.0%. The exception is line 15903, which is resistant to the aphid population and protected by one dominant gene. The high resistance of other lines against a part of the natural population of *S. graminum* is also under oligogenic control. Lines 15600 and 16190 each have one dominant resistance gene, and line 28129 is protected by two genes, the dominant and recessive ones. A recessive resistance gene is presumably present in line 15600. Lines 16237/1 and 16237/2, isolated from the same collection accession, each have one dominant gene effective against individual aphid clones. The loss of effectiveness of distinctly manifested resistance genes causes the expression of previously masked genes with a weak phenotypic manifestation, which differentially interact with insect genotypes.

## 1. Introduction

In recent decades, knowledge of the world’s plant genetic resources has been given particular importance due to the strategic significance of the problem. The main direction of increasing the productivity of agricultural plants, including barley, will be the creation of fundamentally new economical breeding technologies based on the intensive use of plant genetic resources and increasing the ability of plants to withstand biotic and abiotic stressors.

Significant damage to barley (*Hordeum vulgare* L.) crops in the main grain-producing regions of South Russia can be caused by the greenbug, *Schizaphis graminum* Rondani. The insect overwinters on winter and wild cereals; in spring and early summer, it damages small grains; and in June, mass migrations to sorghum seedlings occur. The greenbug is an oligophagous insect; the most preferred crop is barley, while the least preferred are rye [1] and rice [2]. *S. graminum* infestation of barley causes changes in plant metabolism similarly to the drought effects [3]. Even with a relatively low insect population, a significant yield loss can be observed. The phytophage causes the most significant damage to winter and spring crops when migrating to fields during germination [4]. Cultivation of resistant varieties is considered an effective and environmentally friendly way to control this insect. Unfortunately, the specificity of interaction with barley genotypes inherent in *S. graminum* often leads to the loss of the effectiveness of resistance genes. Cultivation over large areas of genetically homogeneous varieties can lead to huge yield losses.

Only five alleles of three genes are known to control the resistance of barley to certain biotypes of the greenbug in the United States. The dominant resistance allele localized on chromosome 1, which was designated *Rsg1a* and later *Rsg1*, was identified in Omugi, Dobaku, Derbent, Kearney, Will, and Post cultivars [5,6,7,8]. Using molecular markers, the *Rsg1* locus was mapped on the long arm of chromosome 3H within a cluster that contains *R* genes encoding nucleotide bunding site leucine-rich repeat (NBS-LRR) proteins [9]. A dominant *Rsg2b* allele was detected in the Pakistani local barley accession PI 426756 [10]. This gene, unlike *Rsg1a*, is expressed against the TX1 biotype. The specific interaction between the host and the phytophage served as a basis for the renaming of resistance genes, for which the symbols *Rsg1* and *Rsg2* were proposed [11,12].

The complexity of the *Rsg1* and *Rsg2* loci is shown. Thus, the accession WBDC336 (PI 682028) of *H. vulgare* ssp. *spontaneum* collected in Turkmenistan is protected by the *Rsg1.a3* allele, which provides resistance to *S. graminum* C, E, H, I, WY81, WY12 MC, and WY86 biotypes [13,14]. It was found that the accession WBDC053 (PI 681777) of *H. vulgare* ssp. *spontaneum* from Pakistan carries the *Rsg2.a3* allele, either closely linked with or allelic to *Rsg2*. Accession WBDC053 is resistant to aphid biotypes B, C, E, I, TX1, WY4A, WY4B, WY81, WY12MC, and WY86, but is severely damaged by the biotypes F, H, WY10MC, and WY10B [15]. The genome interval harboring allele *Rsg1.a3* covers 19 genes of diverse function, with three of them encoding leucine-rich repeat (LRR)-containing proteins [14]. Fifteen genes with LRR protein domains were identified as potential candidates for a new allele of *Rsg2.a3* in the *Rsg2* locus [15].

The *Rsg3* gene, identified in barley landrace PI 565676 from China, is located on the long arm of chromosome 3H. This gene provides high resistance to the *S. graminum* F biotype and moderate resistance to the E biotype. A gene encoding a leucine-rich repeat-containing protein was identified as a putative candidate for the gene underlying *Rsg3* [16]. In addition to PI 565676, three more accessions from China (PI 565657, PI 566459, and CI 2548) are resistant to a number of insect biotypes in the United States [17].

The stock of effective resistance genes can be significantly replenished with landraces and ancient varieties of barley, primarily from Asian countries. One of the main centers for origin of cultivated barley is considered to be the countries of East Asia, where endemic forms inherent only to this region are found. A large number of naked, furcate, awnless, and short-awned forms are concentrated there [18]. It is also known that the mountainous regions of the Indian and Manchurian-Chinese subregions are the centers of origin for most aphid groups [19].

In our experiments, a high frequency of weakly damaged accessions is typical for barley from the countries of East and South Asia [20]. It was shown that barley accessions VIR-3885, VIR-3904, and VIR-4080 from Mongolia have one resistance gene each, different from each other and from the *Rsg1* allele [21].

It should be noted that the screening of the barley collection accessions from East Asia using the Krasnodar aphid population was carried out in 2002–2004. A re-evaluation of accessions from Mongolia in 2021 showed that the resistances to the “old” and “new” populations of *S. graminum* generally coincide, although in 2021, a larger number of accessions were noted in which plants with high resistance to the pest were identified [21]. The ascertainment of the resistance level of barley from other East Asian countries to the “new” insect population 20 years later is of particular interest. The aim of the present work was also to study the effectiveness and genetic control of aphid resistance in barley accessions selected in previous experiments.

## 2. Results

Screening of barley collections from East Asian countries made it possible to identify 40 heterogeneous accessions, in which plants with high (1–4 points) and moderate (5–8 points) resistance to *S. graminum* were identified. Most of the isolated forms are landraces from China (Table 1). In some cases, plants of the accession were clearly differentiated into two phenotypic classes (e.g., in VIR-11200, VIR-15855, and VIR-16200); however, accessions characterized by a wide range of plant damage degrees predominated. Significant variability of the trait may be due to the presence in the aphid population of clones that differ in virulence from barley accessions and/or the expression of genes with a weak phenotypic manifestation.

The results of estimating the resistance of barley accessions collected from the *S. graminum* “old” and “new” populations do not always coincide (Table 1). In 2022, a smaller number of accessions were noted in which plants with high resistance to the pest were identified. In 2022, accessions VIR-12294, VIR-15580, VIR-16104, VIR-16290, VIR-16310, VIR-16312, VIR-16330, VIR-18523, VIR-18526, and VIR-25654, heavily damaged in 2002–2004, contained plants with clearly expressed resistance, and, conversely, previously isolated accessions VIR-12272, VIR-15790, VIR-15903, VIR-16227, VIR-16277, VIR-16292, and VIR-16323 were susceptible in 2022. Apparently, the phenotypic class of resistant genotypes in the studied seed samples was absent, or its frequency was very low. The loss of resistance may also be due to a change in the structure of the pest population and the accumulation of clones that are virulent to previously resistant accessions.

The infestation of 11 pure lines, isolated from heterogeneous accessions in 2004, with a mixture of clones (population) of aphids in 2022 caused, in most cases, severe damage to plants (Table 2). Obviously, the loss of efficiency of resistance 20 years later is due to the accumulation of virulent clones in the *S. graminum* population. The frequency of virulent clones varied from 60.4% (line 15600) to 98% (line 11602). The exception is line 15903 (plant damage 1–2 points, 26.9% of virulent clones). The original accession (VIR-15903) was susceptible to the insect population in 2022 (Table 1), that is, the studied seed sample did not contain a resistant genotype. 

To test the assumption that the selected lines may have genes for greenbug resistance with a weak phenotypic manifestation, we studied the resistance of five moderately resistant lines (variation in plant damage of 4–10 points) to the randomly selected 30 clones isolated from the Krasnodar population of *S. graminum*. A distinctly expressed resistance was observed in four accessions; moderate resistance was estimated at 5–8 points in all lines; and both types of resistance were being manifested only against some aphid clones (Table 3).

As a result of the resistance assessment of 11 barley lines and cultivar Post to all 108 clones of *S. graminum*, 52 insect virulence phenotypes (biotypes) were identified. The phenotype avirulent to line 15903 and strongly damaging the rest of the accessions was dominating (20.4%) (Table 4); 38 phenotypes were encountered with a low frequency (0.9%).

Greenbug clones differing in virulence phenotypes (“test clones”) make it possible to identify genes of resistance to the phytophage in the isolated barley accessions. If the only clone avirulent to a tester for a given resistance gene severely damages an accession being evaluated, this indicates that the accession does not have a functional allele for that gene. In our experiments, 11 *S. graminum* clones were sufficient to identify the resistance alleles (Table 5).

Thus, lines 11602, 15903, and 26319 were resistant to clone 1, while 8 lines and cultivar Post were susceptible. Therefore, the resistance alleles in the three lines differ from the alleles that the rest of the accessions have. The interaction between the studied accessions and *S. graminum* with virulence phenotype 2 indicates a difference in the genetic control of the resistance of lines 12272 and 15903 and other accessions. Further comparison of the resistance of accessions to the test clones 3–11 indicates that all 11 lines have different alleles of resistance to the greenbug, which differ from the previously identified *Rsg1*.

Under laboratory conditions, we analyzed the segregation of the F_2_ hybrids obtained by crossing the susceptible cultivar Belogorsky with line 15903, which had an effective gene(s) of resistance to *S. graminum*, as well as with lines 15600, 16190, and 28129, which we used as differentials in population genetic studies. Hybrids were infested with aphid clones that were avirulent to resistant accessions.

At the time of evaluating the segregation of F_2_ hybrids, the damage to resistant accessions and F_1_ hybrids corresponded mainly to 1–3 points, that is, resistance dominated in all cross combinations. The ratio of resistant (R) and susceptible (S) phenotypes 135:45 in the F_2_ (Belogorsky × 16190) corresponded to the assumption of monogenic dominant inheritance of the trait (χ^2^ = 0.03; *p* = 0.80–0.90) (Table 6). In combinations of crossings of the cultivar Belogorsky with lines 15600 and 15903, the ratios of phenotypes satisfied the assumption of monogenic dominant control of the trait, although the presence of recessive genes is not excluded. Line 28129 has dominant and recessive genes that are effective against individual aphid clones. The hypothesis of monogenic control of the trait is rejected (χ^2^ = 8.18; *p* < 0.05). The presence of a recessive gene was confirmed in the F_3_ Belogorsky × 28129, where families segregating at a ratio of 1:3 were identified.

Lines 16237/1 and 16237/2, isolated from the collection accession VIR-16237, have different alleles of resistance to the greenbug, which differ from the previously identified *Rsg1* allele (Table 5). At the same time, genes identified by using test clones may have different alleles of the same gene. We analyzed the F_2_ 16237/1 × 16237/2 hybrid under infestation with a clone avirulent to both lines. Segregation for two dominant genes (62R:7S; χ^2^_15:1_ = 1.79; P = 0.10–0.20) indicates the presence of non-allelic resistance genes in these accessions. 

## 3. Discussion

The genetic diversity of barley accessions from East Asian countries in terms of greenbug resistance is quite high. In 2022, among 778 accessions from China, Japan, and Nepal, 40 heterogeneous accessions were identified, among which plants with high resistance to *S. graminum* were revealed. The other 30 heterogeneous accessions containing plants with high resistance were identified earlier, in the experiments of 2002–2004. The richest source of replenishment of the gene bank of resistance to a dangerous pest is barley landraces from China.

The high frequency of *S. graminum*-resistant accessions among barley landraces from China is indicative of a long-standing host–consumer relationship. N.I. Vavilov pointed out that “... immunity is developed under the influence of natural selection only under those conditions that contribute to the development of infection, and, as a rule, are detected only where one or another parasite is present, against which selection produces immunity” [22]. 

Experiments with insect clones showed that a wide variation in the degree of plant damage is primarily due to the presence of clones with different virulences for the studied barley accessions in the Krasnodar population. As a result of the resistance assessment of 11 lines isolated from heterogeneous barley accessions to the 108 *S. graminum* clones, 52 insect virulence phenotypes were identified. Although all lines have resistance alleles that differ from each other and from the previously identified *Rsg1* (Post cultivar), their efficiency is low. The frequency of clones virulent to ten lines and cultivar Post varies from 60.4% to 98.0%. The exception is line 15903, highly resistant to the aphid population, which is protected by one dominant gene. We were also able to identify a low level of plant resistance to individual greenbug clones.

It should be noted that there was a striking change in the Krasnodar insect population in 2022 compared to the previous year: in 2021, only 41.4% of clones in the population were virulent to the Post cultivar [21]. Earlier, during population genetic studies, the frequency of clones virulent to Post in June 2013 was 26–36%, to line 15600 was 44–50%, to line 16190 was 18%, and to line 28129 was 30–34% [23].

Earlier, we noted the high polymorphism of the North Caucasian populations in terms of virulence to accessions of barley, oats, and sorghum. Unexpectedly, the variability of the population of *S. graminum* turned out to be very high in Dagestan, where grain crops occupy relatively small areas, most of the cereal weeds usually “burn out” in the first half of summer due to high temperatures, the number of phytophages in the fields is usually very small, and the main host plant is wild perennial sorghum [21,23]. In the Krasnodar Territory and other regions of Russia, where barley occupies vast areas, only varieties susceptible to the insect are cultivated, which does not contribute to the formation and selection of aphid phenotypes with specific virulence. Among the 248 barley cultivars included in the State Register of Breeding Achievements approved for use in Russia, only 11 cultivars have plants with distinct resistance to the greenbug [24]. Obviously, the virulence phenotypes (biotypes) of the aphid in the North Caucasus are formed only when they feed on wild grasses. The important role of wild host plants in the evolution of *S. graminum*, including the emergence of biotypes with specific virulence for cultivated cereals, was noted earlier [12,25,26,27].

We evaluated the differential interaction of *S. graminum* genotypes with clearly manifested resistance genes (plant damage up to 4 points) in barley accessions. At the same time, when the lines were infested with insect clones, three phenotypic classes were noted: resistant (plant damage at the level of 1–4 points, the action of genes with high expressivity in this accession to this clone), moderately resistant (5–8 points, expression of weakly manifested genes), and susceptible (9–10 points, virulence to both major and minor resistance genes). Thus, not only clearly manifested but also weakly expressed (minor) genes of barley resistance to the aphid interact differentially with phytophage genotypes and cannot be considered the basis for durable resistance. Future analysis of allelic relationships among the resistance genes of the studied accessions and the known *Rsg* alleles will help extend our knowledge of the genetic diversity of a representative group of East Asian barley landraces from the VIR collection.

## 4. Materials and Methods

### 4.1. Plant Materials

The material for the experiments comprised 778 accessions of barley, mainly landraces; entries from the collection of the N.I. Vavilov All-Russian Institute of Plant Genetic Resources (VIR) from China (313 accessions), Japan (450), and Nepal (15); as well as the control susceptible cultivar Belogorsky. In addition, we studied resistance to *Schizaphis graminum* of cultivar Post (a carrier of the *Rsg1* gene) and 11 pure homozygous lines isolated from heterogeneous accessions VIR-11602, VIR-12272, VIR-15600, VIR-15903, VIR-16190, VIR-16237, VIR-16291, VIR-18455 (China), VIR-26319 (Nepal), and VIR-28129 (North Korea). Two lines, 16237/1 and 16237/2, differing in morphological traits, were selected from the VIR-16237 accession. The resistant plants of these accessions were brought to maturity, the seeds were multiplied, and the obtained lines were again estimated for resistance to the greenbug. Segregation for aphid resistance of F_2_ hybrids from crossing Belogorsky cultivars with lines 15600, 15903, 16190, and 28129, as well as that of F_2_ 16237/1 × 16237/2 and 60 families of F_3_ Belogorsky × 28129, was analyzed.

### 4.2. Insect

The damage to the studied barley accessions by the Krasnodar (Kuban Experimental Station of the Vavilov Institute of Plant Genetic Resources) insect population and clones isolated from it was assessed. *S. graminum* was collected in July 2022 on sorghum (*Sorghum bicolor* (L.) Moench) varieties Kubanskoe Krasnoe 1677, Efremovskoe Beloe, and SLV-2, which were sown along the perimeter of the experimental field. The collected samples were cloned in the laboratory. To do this, the seeds of the Belogorsky cultivar were laid out on cotton wool moistened with water and placed in halves of Petri dishes. After 3–5 days, a female was placed on the emerging shoots in each dish and covered with glasses, the upper part of which was covered with mesh cloth. After the emergence of the larvae, the cages with the resulting clones were maintained in the light chamber at 21 ± 1 °C and 16:8 h photoperiod under ‘‘cool-white’’ fluorescent lighting. Further maintenance of clones was carried out by shaking off aphids into similar cages [21]. The experiments maintained 108 insect clones.

### 4.3. Plant Resistance Evaluation

When evaluating resistance, the experimental accessions were sown in rows in plastic trays with soil. Each tray contained 10 rows of tested accessions, two rows of the nonresistant control, and Post cultivar. Juvenile plants were infested with an insect population (a mixture of 108 clones at our disposal) by shaking aphids of different ages onto barley (4–5 individuals per plant). Plant tissues necrotize in the places of greenbug feeding, which makes it possible to assess plant damage at these points. Plant damage was assessed on a scale: 0—no damage, 1—1–10% of the leaf surface damaged, 2—11–20% … 10—90–100%, plant death. Damage ratings 1–4 (from 1–10 to 31–40% of leaf area damaged) were referred to as resistance (R) and ratings of 9–10 as susceptibility (S). The accessions identified as resistant were retested.

The resistance of cultivar Post and 11 lines isolated from heterogeneous accessions to 108 *S. graminum* clones was assessed. Experimental accessions and susceptible control were sown in a circle in vessels filled with soil and covered with glasses. The seedlings that emerged were infested with aphids of one clone, and when cultivar Belogorsky died, plant damage was assessed according to the above-mentioned scale. If the trait manifestation was not clear, the experiment was repeated.

To analyze the segregation in terms of resistance to aphids of F_2_ hybrids and F_3_ families from crossing-resistant lines with a susceptible tester, one row of P_1_, P_2_, and F_1_ and 7–8 rows of F_2_ or F_3_ were sown in a tray with soil. F_2_ seeds were collected from one F_1_ plant, and each F_3_ family was the progeny of one F_2_ plant. Juvenile plants were infested with an aphid clone avirulent to the resistant line, and when the resistant parent died (9–10 points), plant damage was assessed. Susceptible (S) plants were considered to be similar in the degree of damage to the cultivar Belogorsky.

The resistant class (R) included plants similar to the paternal form but slightly more damaged (suggested heterozygotes). When studying the allelic relations of resistance genes, the segregation in F_2_ (16237/1 × 16237/2) was analyzed upon the death of the susceptible Belogorsky control, which was sown together with P_1_, P_2_, and F_1_. The compliance of the observed ratio with the expected one was checked using the χ^2^ test.

## 5. Conclusions

The results of the experiments indicate a high diversity of barley accessions from the countries of East Asia in genes of greenbug resistance. However, the polymorphism of the natural insect population in terms of virulence to barley accessions is also very high. Barley accessions, which are characterized by a significant variation in the degree of plant damage, have resistance genes that are effective against a larger (in line 15903) or smaller (in lines 18455, 12272, and others) part of the natural population of *S. graminum*.

Line 15903, highly resistant to the aphid population, is protected by one dominant gene, and it can be used directly in breeding programs. The high level of resistance of other lines against a part of the natural population of *S. graminum* is also controlled by oligogenes. Thus, lines 15600 and 16190 each have one dominant resistance gene, and line 28129 is protected by two genes, the dominant and recessive ones. In line 15600, the presence of a recessive resistance gene is not excluded. Lines 16237/1 and 16237/2, isolated from one collection accession, each have one dominant gene effective against individual aphid clones.

The resistant lines that we have isolated, in addition to genes with a distinct phenotypic manifestation, also have weakly expressed genes of resistance to *S. graminum*, which manifest themselves in cases of loss of effectiveness of the major gene. Weakly expressed (minor) genes of barley resistance to the aphid, as well as clearly manifested ones, interact differentially with phytophage genotypes and cannot be considered the basis for durable resistance.

Obviously, further, closer study of landraces collected during the first VIR expeditions will help to broaden the genetic diversity of cultivated barley varieties in terms of resistance to a dangerous pest.

## Figures and Tables

**Table 1:** Barley accessions from East Asia, identified for their greenbug resistance.

VIR Catalogue Number	Accession	Variety	Origin	Damage Score	
				2002–2004	2022
10961	Shiromugi N1 (Kinai)	*parallelum*	Japan	ND	4, 7, 10
10980	Hanbozu	*japonicum*	Japan	ND	3, 4, 8
10984	Koshimaki N23 (Miyagi-ken)	*parallelum, pallidum*	Japan	ND	2, 3, 7
11009	Bizen-wase N53 (Fukushima-ken)	*subparallelum*	Japan	ND	3, 4, 7
11126	Kairyo-omugi	*parallelum*	Japan	ND	2, 3, 5, 7
11162	Local	*subpyramidatum*	Japan	ND	3, 7, 10
11200	Local	*pyramidatum, brachyathrum*	Japan	ND	4, 10
20247	Aizu N4	*parallelum*	Japan	ND	4, 10
21341	Jamatohadaka	*subnudipyramidatum*	Japan	ND	4, 10
22429	Azumamugi	*subpyramidatum*	Japan	ND	3, 4, 10
25585	Roкulеn	*pallidum*	Japan	ND	3, 7, 10
11602	Local	*pallidum*	China	2, 3, 10	3, 4, 8–10
12237	Chekiang	*pallidum*	China	ND	3, 4, 8
12272	Shantung	*coeleste*	China	3–10	9, 10
12294	Local	*pallidum*	China	9, 10	2, 3, 7
15578	Local	*pallidum*	China	2, 4, 10	ND
15600	Local	*nigrum*	China	6, 7, 10	ND
15580	Local	*hangaicum*	China	9–10	4, 7, 8
15789	Local	*pallidum*	China	7–10	4, 10
15790	Local	*nigritonsum*	China	4–8, 10	10
15855	Local	*pallidum*	China	1, 8–10	2, 10
15903	Local	*horsfordianum*	China	3, 9, 10	10
16104	Local	*pallidum*	China	10	4, 9, 10
16106	Local	*sinicum*	China	4-10	4, 9, 10
16109	Local	*pallidum*	China	4, 10	ND
16111	Local	*breviaristatum*	China	1–8	ND
16118	Local	*pallidum*	China	2–6, 10	2, 4, 10
16142	Local	*pallidum*	China	3, 10	ND
16155	Local	*breviaristatum*	China	3–5, 7, 9, 10	ND
16156	Local	*pallidum*	China	ND	2, 3, 10
16175	Local	*breviaristatum*	China	2, 3, 10	ND
16178	Local	*breviaristatum*	China	3, 7–10	ND
16179	Local	*pallidum*	China	2, 3, 10	ND
16187	Local	*hypatherum*	China	2–4, 10	ND
16190	Local	*breviaristatum*	China	3–5, 9, 10	ND
16193	Local	*breviaristatum*	China	2, 3, 9, 10	ND
16199	Local	*pallidum*	China	3, 4, 9, 10	ND
16200	Local	*breviaristatum*	China	2–4, 10	1, 10
16201	Local	*pallidum*	China	2, 4, 9, 10	ND
16204	Local	*pallidum*	China	4, 10	ND
16212	Local	*pallidum*	China	3, 4, 9, 10	ND
16219	Local	*breviaristatum*	China	2–4, 9, 10	ND
16220	Local	*breviaristatum*	China	3–6, 10	2–4, 8, 10
16222	Local	*breviaristatum*	China	4–6, 10	ND
16223	Local	*pallidum*	China	4–6, 10	ND
16225	Local	*pallidum*	China	5, 7, 9, 10	2, 10
16227	Local	*pallidum*	China	3, 10	10
16237	Local	*breviaristatum*	China	3, 4, 7, 9, 10	2, 7, 8, 10
16241	Local	*breviaristatum*	China	3, 4, 10	ND
16246	Local	*breviaristatum*	China	3–6, 9, 10	ND
16247	Local	*pallidum*	China	4, 5, 9, 10	ND
16248	Local	*pallidum*	China	2–6, 8, 9, 10	ND
16251	Local	*pallidum*	China	2–7, 9, 10	ND
16268	Local	*pallidum*	China	2, 3, 10	ND
16270	Local	*pallidum*	China	4, 10	ND
16277	Local	*breviaristatum*	China	4, 10	10
16288	Local	*pallidum*	China	3, 10	ND
16290	Local	*pallidum*	China	10	4, 10
16291	Local	*breviaristatum*	China	2, 10	ND
16292	Local	*hypatherum*	China	3, 4, 10	10
16295	Local	*pallidum*	China	2, 3, 6–10	ND
16297	Local	*pallidum*	China	2–4, 9, 10	ND
16310	Local	*pallidum*	China	10	4, 10
16312	Local	*pallidum*	China	9, 10	4, 8, 10
16322	Local	*pallidum*	China	8–10	4, 10
16323	Local	*pallidum*	China	3–7, 9, 10	10
16329	Local	*breviaristatum*	China	4–10	3, 4, 8, 10
16330	Local	*breviaristatum*	China	9, 10	4, 10
16331	Local	*breviaristatum*	China	3–6, 9, 10	ND
16338	Local	*breviaristatum*	China	5–7, 9, 10	2, 4, 10
18439	Local	*himalayense*	China	7–10	3, 7, 8, 10
18445	Local	*violaceum, pallidum, coeleste, himalayense*	China	ND	4, 8, 10
18455	Local	*coeleste, himalayense, tibetanum*	China	2, 3, 9, 10	4, 8, 10
18523	Peasant	*pallidum*	China	10	4, 8–10
18526	Si-chan-ben-di-da-mai	*pallidum*	China	10	4, 8, 10
18968	Bai-ding-ching-ko-165	*coeleste, himalaense, violaceum*	China	ND	3, 7, 10
18992	Ti 162	*tibetanum*	China	ND	4, 10
25654	NB 4401	*pallidum*	Nepal	10	3, 4, 7
26319	NB 6905	*pallidum*	Nepal	3, 10	ND
28129	Namji Milyang Native K647 1007	*hypatherum*	North Korea	2	ND
22089	Belogorsky (control)		Russia	9, 10	9, 10

Note: ND—no data.

**Table 2:** Resistance of barley lines to the Krasnodar population of *Schizaphis graminum*.

Accession	Origin	Frequency of Virulent Clones, %	Resistance to the Population, Score
11602	China	98.0	7–10
12272	China	94.8	9, 10
15600	China	60.4	5, 7–9
15903	China	26.9	1, 2
16190	China	60.9	5, 7, 9
16237/1	China	97.6	7–10
16237/2	China	92.5	8–10
16291	China	97.4	9, 10
18455	China	89.3	7–10
26319	Nepal	74.6	4, 7, 9, 10
28129	North Korea	80.8	4, 5, 7, 8, 10
Post (control)		83.0	7–9
Belogorsky (control)		100	9, 10

**Table 3:** Resistance of barley lines to 30 greenbug clones.

Accession	Resistance to the Population, Score	The Frequency of Clones That Cause Different Levels of Resistance (1–10 Points)		
		1–4	5–8	9–10
15600	5, 7–9	0.27	0.10	0.63
16190	5, 7, 9	0.23	0.03	0.74
18455	7–10	-	0.20	0.80
26319	4, 7, 9, 10	0.03	0.27	0.70
28129	4, 5, 7, 8, 10	0.10	0.13	0.77

**Table 4:** Phenotypic diversity of the Krasnodar greenbug population.

Virulence Phenotype	Number of Phenotypes	Resistance of Barley Lines to Greenbug Phenotypes for Virulence for Clearly Manifested Genes											
		Post	16190	28129	15600	18455	16237/1	16237/2	11602	12272	15903	16291	26319
1	22	S	S	S	S	S	S	S	S	S	**R**	S	S
2	9	S	S	S	S	S	S	S	S	S	S	S	S
3	9	S	S	S	S	S	S	S	S	S	**R**	S	**R**
4	4	S	**R**	S	**R**	S	S	S	S	S	**R**	S	S
5	4	S	**R**	S	**R**	S	S	S	S	S	S	S	S
6	4	S	**R**	**R**	**R**	S	S	S	S	S	S	S	S
7	3	S	**R**	**R**	**R**	S	S	S	S	S	**R**	S	S
8	3	**R**	**R**	R	**R**	S	S	S	S	S	**R**	S	**R**
9	2	S	S	S	S	S	S	**R**	S	S	**R**	S	S
10	2	**R**	S	S	**R**	S	S	S	S	S	**R**	S	S
11	2	S	S	S	**R**	S	S	S	S	S	**R**	S	S
12	2	S	**R**	S	S	S	S	S	S	S	**R**	S	S
13	2	S	S	S	S	**R**	S	S	S	S	**R**	S	S
14	2	**R**	**R**	S	**R**	S	S	S	S	S	**R**	S	S
15	1	S	S	S	S	S	S	S	S	S	**R**	**R**	S
16	1	**R**	S	S	S	S	S	S	S	**R**	S	S	S
17	1	S	S	S	S	S	S	S	S	**R**	**R**	S	S
18	1	**R**	S	S	S	S	S	S	S	S	**R**	S	S
19	1	S	S	S	**R**	S	**R**	S	S	S	**R**	S	S
20	1	**R**	S	S	**R**	S	S	S	S	**R**	**R**	**R**	S
21	1	S	**R**	**R**	S	S	S	S	S	S	S	S	S
22	1	S	**R**	S	S	S	S	S	S	S	S	S	S
23	1	S	S	**R**	**R**	S	S	S	S	S	S	S	S
24	1	S	S	S	**R**	S	S	S	S	S	S	S	S
25	1	**R**	**R**	**R**	**R**	S	S	**R**	S	**R**	**R**	S	S
26	1	S	**R**	S	**R**	**R**	**R**	**R**	S	S	**R**	S	S
27	1	R	**R**	S	S	S	**R**	S	S	S	**R**	S	S
28	1	S	S	S	S	S	S	S	**R**	S	**R**	S	**R**
29	1	S	S	**R**	S	S	S	S	S	S	**R**	S	**R**
30	1	**R**	**R**	S	S	S	S	S	S	S	**R**	S	S
31	1	S	**R**	S	S	**R**	S	S	S	S	**R**	S	S
32	1	S	S	S	**R**	**R**	S	S	S	S	S	S	S
33	1	S	S	**R**	**R**	S	S	S	S	S	**R**	S	**R**
34	1	S	**R**	**R**	**R**	S	S	**R**	S	S	**R**	S	S
35	1	**R**	**R**	**R**	**R**	**R**	S	S	S	S	**R**	S	S
36	1	S	**R**	S	**R**	S	S	S	S	**R**	**R**	S	S
37	1	S	**R**	R	S	**R**	S	S	S	S	S	S	**R**
38	1	S	**R**	S	S	S	S	**R**	S	S	**R**	S	S
39	1	S	S	S	S	**R**	S	S	S	S	S	S	**R**
40	1	**R**	**R**	S	**R**	S	S	**R**	**R**	**R**	**R**	S	S
41	1	**R**	**R**	S	**R**	S	S	**R**	S	S	**R**	S	S
42	1	S	S	S	S	**R**	S	S	S	S	S	S	S
43	1	**R**	**R**	**R**	**R**	S	S	S	S	S	S	S	S
44	1	S	S	S	**R**	**R**	S	S	S	S	S	S	S
45	1	S	**R**	S	**R**	S	S	S	S	S	**R**	S	**R**
46	1	S	**R**	**R**	**R**	S	S	S	S	S	**R**	S	**R**
47	1	S	S	S	S	**R**	S	S	S	S	**R**	S	**R**
48	1	S	S	S	S	S	S	S	S	S	**R**	**R**	**R**
49	1	S	**R**	S	S	S	S	S	S	S	S	S	**R**
50	1	**R**	**R**	S	**R**	S	S	S	S	S	**R**	S	**R**
51	1	S	**R**	S	S	**R**	S	S	S	S	S	S	**R**
52	1	S	S	S	S	S	S	S	S	S	S	S	**R**

Note: Here and after **R**—resistance of accession (plant damage 0–4 points), S—susceptibility (plant damage 5–10 points).

**Table 5:** Resistance of barley accessions to test clones of *Schizaphis graminum*.

Accession	Plant Resistance to the Aphid Clone										
	1	2	3	4	5	6	7	8	9	10	11
11602	**R**	S	S	S	S	S	S	S	S	S	S
12272	S	**R**	S	S	S	S	S	S	S	S	S
15600	S	S	**R**	S	S	**R**	S	S	S	S	S
15903	**R**	**R**	S	**R**	**R**	S	**R**	**R**	**R**	S	S
16190	S	S	S	S	**R**	S	S	S	S	S	**R**
16237/1	S	S	S	S	S	**R**	S	S	S	S	S
16237/2	S	S	S	S	S	S	**R**	S	S	S	S
16291	S	S	S	S	S	S	S	**R**	S	S	S
18455	S	S	S	S	S	S	S	S	**R**	S	S
26319	**R**	S	S	**R**	S	S	S	S	S	**R**	S
28129	S	S	S	S	S	S	S	S	S	S	**R**
Post (*Rsg1*)	S	S	S	S	S	S	S	S	S	S	S

**Table 6:** Segregation for resistance to *S. graminum* of the F_2_ hybrids from crossing resistant barley accessions and a susceptible tester.

Cross Combination	Total Number of Plants	The Ratio of Phenotypes (R:S)		χ^2^	*p*
		Observed	Expected		
Belogorsky × 16190	180	135:45	3:1	0.03	0.80–0.90
Belogorsky × 28129	458	370:88	3:1	8.18	<0.05
			13:3	0.06	0.75–0.80
Belogorsky × 15600	358	280:78	3:1	1.97	0.10–0.20
			13:3	2.17	0.10–0.20
Belogorsky × 15903	262	203:59	3:1	0.86	0.25–0.50
			13:3	2.44	0.10–0.20

Note: χ^2^_0.05_ = 3.84.

## Data Availability

Data are contained within the article.

## References

[1] Schweissing F., Wilde G. (1979). Predisposition and nonpreference of greenbug for certain host cultivars. Environ. Entomol..

[2] Wilson R.L., Starks K.J. (1981). Effect of culture-host preconditioning on greenbug response to different plant species. Southwest. Entomol..

[3] Cabrera H.M., Argandoňa V.H., Zúňiga G.E., Corcuera L.J. (1995). Effect of infestation by aphids on the water status of barley and insect development. Phytochemistry..

[4] Pike K.S., Schaffner R.L. (1985). Development of autumn populations of cereal aphids, *Rhopalosiphum padi* (L.) and *Schizaphis graminum* (Rondani) (Homoptera: Aphididae) and their effects on winter wheat in Washington state. J. Econ. Entomol..

[5] Gardenhire J.H., Chada H.L. (1961). Inheritance of greenbug resistance in barley. Crop Sci..

[6] Smith O.D., Schlehuber A.M., Curtis B.C. (1962). Inheritance studies of greenbug (*Toxoptera graminum* Rond.) resistance in four varieties of winter barley. Crop Sci..

[7] Gardenhire J.H., Tuleen N.A., Stewart K.W. (1973). Trisomic analysis of greenbug resistance in barley, *Hordeum vulgare* L.. Crop Sci..

[8] Edwards L.H., Smith E.L., Pass H., Morgan G.H. (1985). Registration of Post barley. Crop Sci..

[9] Azhaguvel P., Mornhinweg D., Vidya-Saraswathi D., Rudd J.C., Chekhovskiy K., Saha M., Close T.J., Dahleen L.S., Weng Y. (2014). Molecular mapping of greenbug (*Schizaphis graminum*) resistance gene *Rsg1* in barley. Plant Breed..

[10] Merkle O.G., Webster J.A., Morgan G.H. (1987). Inheritance of a second source of greenbug resistance in barley. Crop Sci..

[11] Puterka G.J., Peters D.C., Kerns D.L., Slosser J.E., Bush L., Worrall D.W., McNew R.W. (1988). Designation of two new greenbug (Homoptera: Aphididae) biotypes G and H. J. Econ. Entomol..

[12] Anstead J.A., Burd J.D., Shufran K.A. (2003). Over-summering and biotypic diversity of *Schizaphis graminum* (Homoptera: Aphididae) populations on noncultivated grass hosts. Environ. Entomol..

[13] Armstrong J.S., Mornhinweg D.W., Payton M.E., Puterka G.J. (2016). The discovery of resistant sources of spring barley, *Hordeum vulgare* ssp. spontaneum, and unique greenbug biotypes. J. Econ. Entomol..

[14] Xu X., Mornhinweg D.W., Bai G., Steffenson B., Bian R., Li G., Bernardo A. (2021). *Rsg1.a3*: A new allele conferring unique resistance to greenbug Biotype H at the *Rsg1* locus in *Hordeum vulgare* ssp. spontaneum. Crop Sci..

[15] Xu X., Mornhinweg D., Bernardo A., Li G., Bian R., Steffenson B.J., Bai G. (2022). Characterization of *Rsg2.a3*: A new greenbug resistance allele at the *Rsg2* locus from wild barley (*Hordeum vulgare* ssp. spontaneum). Crop J..

[16] Xu X., Mornhinweg D., Bai G., Li G., Bian R., Bernardo A., Armstrong J.S. (2023). Characterization of *Rsg3*, a novel greenbug resistance gene from the Chinese barley landrace PI 565676. Plant Genome.

[17] Mornhinweg D., Armstrong J.S., Puterka G.J. (2017). New greenbug resistant sources in winter barley, *Hordeum vulgare*. Southwest. Entomol..

[18] Vavilov N.I. (1965). Selected Works.

[19] Shaposhnikov G.K. (1967). Aphid Evolution in Connection with Specialization and Changes of Hosts. D. Sc. Thesis.

[20] Radchenko E.E., Kuznetsova T.L., Zveinek I.A., Kovaleva O.N. (2014). Greenbug resistance in barley accessions from East and South Asia. Russ. Agric. Sci..

[21] Radchenko E.E., Abdullaev R.A., Akimova D.E., Zajtseva I.Y. (2022). Genetic diversity of barley accessions from Mongolia for greenbug resistance. Ecol. Genet..

[22] Vavilov N.I. (1964). Selected Works.

[23] Radchenko E.E., Alpatieva N.V., Chumakov M.A., Abdullaev R.A. (2019). Variability of the North Caucasian populations of the greenbug for host virulence and discovered by molecular markers. Russ. J. Gen..

[24] Abdullaev R.A., Batasheva B.A., Alpatieva N.V., Chumakov M.A., Radchenko E.E., Kovaleva O.N., Yakovleva O.V. (2020). Resistance of barley cultivars approved for use in Russia to harmful organisms and toxic aluminum ions. Proc. Appl. Bot. Gen. Breed..

[25] Porter D.R., Burd J.D., Shufran K.A., Webster J.A., Teetes G.L. (1997). Greenbug (Homoptera: Aphididae) biotypes: Selected by resistant cultivars or preadapted opportunists?. J. Econ. Entomol..

[26] Shufran K.A., Burd J.D., Anstead J.A., Lushai G. (2000). Mitochondrial DNA sequence divergence among greenbug (Homoptera: Aphididae) biotypes: Evidence for host-adapted races. Insect Mol. Biol..

[27] Anstead J.A., Burd J.D., Shufran K.A. (2002). Mitochondrial DNA sequence divergence among *Schizaphis graminum* (Hemiptera: Aphididae) clones from cultivated and non-cultivated hosts: Haplotype and host associations. Bull. Entomol. Res..

